# What is the role of non-surgical clinicians in the assessment and management of degenerative cervical myelopathy? – Insights from the RECODE-DCM peri-operative rehabilitation incubator

**DOI:** 10.1016/j.bas.2025.104275

**Published:** 2025-05-08

**Authors:** Rohil V. Chauhan, Andreas K. Demetriades, Timothy F. Boerger, Justin M. Lantz, Caroline Treanor, Sukhvinder Kalsi-Ryan, Vishal Kumar, Lianne Wood, Joshua Plener, Nicky Wilson, Maryse Fortin, Carlo Ammendolia, Annalena Paus, Rana S. Dhillon, Benjamin Davies, Michael G. Fehlings, David B. Anderson, RECODE-DCM Peri-Operative Rehabilitation Incubator

**Affiliations:** a Auckland Spine Surgery Centre, Auckland, New Zealand; b Active Living and Rehabilitation: Aotearoa New Zealand, Health and Rehabilitation Research Institute, Faculty of Health and Environmental Sciences, Auckland University of Technology, Akoranga Campus, Northcote, Private Bag, 92006, Auckland, New Zealand; c Department of Neurosurgery, University Neurosurgical Center Holland, UMC | HMC | HAGA, Leiden, The Hague, the Netherlands; d Department of Neurosurgery, Royal Infirmary, Edinburgh, UK; e Medical College of Wisconsin, Milwaukee, USA; f Division of Biokinesiology and Physical Therapy, University of Southern California, Los Angeles, CA, USA; g Department of Family Medicine, Keck School of Medicine, University of Southern California, Los Angeles, CA, USA; h National Neurosurgical Centre, Beaumont Hospital, Dublin 9, Ireland; i School of Physiotherapy, Royal College of Surgeons in Ireland (RCSI) University of Medicine and Health Sciences, Dublin 2, Ireland; j KITE Research Institute, University Health Network, Toronto, Canada; k Postgraduate Institute of Medical Education and Research, Chandigarh, India; l Department of Public Health and Sports Science, University of Exeter, Exeter, EX1 2LU, UK; m Department of Medicine, Mount Sinai Hospital, Toronto, Canada; n Physiotherapy Department, King's College Hospital NHS Foundation Trust, UK; o London, UK & Centre for Rheumatic Disease, King's College London, London, UK; p Department of Health, Kinesiology & Applied Physiology, Concordia University, Montreal, Qc, Canada; q Department of Surgery, University of Toronto, Toronto, Canada; r Department of Therapeutic Health Professions, University Hospital Münster, Germany; s Department of Surgery, University of Melbourne, Victoria, Australia; t Department of Neurosurgery, St Vincents Hospital Melbourne, Victoria, Australia; u Division of Neurosurgery, Department of Clinical Neurosciences, University of Cambridge, Cambridge, UK; v Myelopathy.org, Cambridge, UK; w Division of Neurosurgery and Spine Program, University of Toronto and Toronto Western Hospital, Canada; x Sydney School of Health Sciences, Faculty of Medicine and Health, The University of Sydney, Sydney, Australia; y Sydney Spine Institute, Burwood, Sydney, Australia

**Keywords:** Degenerative cervical myelopathy, Non-surgical, Nonoperative, Rehabilitation, Multidisciplinary

## Abstract

**Introduction:**

Evidence on degenerative cervical myelopathy (DCM) has frequently focussed on surgical management, overlooking the role of non-surgical clinicians. Their contributions in the patient journey remain largely underexplored in the literature.

**Research question:**

What is the role of non-surgical clinicians in the assessment and management of people with DCM?

**Material and methods:**

This narrative review synthesizes knowledge from a comprehensive MEDLINE search and the collective expertise of the RECODE-DCM Peri-Operative Rehabilitation Incubator, an expert working group hosted by Myelopathy.org. Key domains of non-surgical clinician involvement include: 1) early recognition and referral, 2) patient education, 3) pain management, 4) preoperative management, and 5) postoperative rehabilitation.

**Results:**

Timely DCM diagnosis depends on first-contact clinicians recognizing hallmark symptoms. In the absence of standardized screening criteria, tools like the modified Japanese Orthopaedic Association score can support early identification. Non-surgical clinicians educate patients with mild or non-myelopathic spinal cord compression to recognize signs of DCM progression, ensuring timely surgical consultation. These clinicians also play a multidisciplinary role in the biopsychosocial management of pain, incorporating pharmacological and non-pharmacological strategies to address nociceptive and neuropathic pain. While predictors of postoperative outcomes, such as disease severity, gait dysfunction and smoking, are known, evidence on preoperative optimization and prehabilitation remains limited. Emerging research highlights the benefits of early postoperative rehabilitation, including cervical range of motion and stabilization exercises, in improving 12-month postoperative outcomes.

**Discussion and conclusion:**

Non-surgical clinicians play an integral role in DCM management across the care continuum. A multidisciplinary, patient-centred approach is essential. Postoperative rehabilitation holds promise, but prospective trials are necessary to establish standardization and optimal strategies for clinical delivery.

## Highlights

• Early DCM recognition by non-surgical clinicians is critical; tools like mJOA can aid screening.
• Educating patients on DCM progression aids timely surgical consultation for mild and NMSCC cases.
• Pain management requires a multidisciplinary biopsychosocial approach to nociceptive and neuropathic pain.
• Preoperative optimization comorbidities, psychology, and cervical musculature and smoking cessation.
• Early postoperative rehabilitation shows promise; prospective trials are needed to define optimal delivery.

## 1 Introduction

Degenerative cervical myelopathy (DCM) is the leading cause of adult spinal cord dysfunction, affecting an estimated 2.3 % of adults globally (Badhiwala et al., 2020; Nouri et al., 2015a). DCM arises from cervical spinal cord compression due to a combination of degenerative and congenital factors, manifesting in debilitating symptoms such as hand dysfunction, neck and arm pain, limb paraesthesia, gait and balance decline and autonomic dysfunction (Jiang et al., 2023). With the aging population projected to grow worldwide, both the prevalence and socioeconomic impact of DCM are expected to increase significantly (Davies et al., 2018b). Although early diagnosis and timely surgical decompression are pivotal for optimal long-term outcomes (Fehlings et al., 2017), these are often delayed, contributing to preventable functional decline (Davies et al., 2018b).

Historically, DCM research has predominantly focused on surgical management, with little attention to the role of non-surgical management and the contribution of non-surgical clinicians (Boerger et al., 2022b). However, non-surgical clinicians, particularly those in the primary healthcare, medical and allied health disciplines, can play a key role in the comprehensive assessment and management of DCM. Their responsibilities encompass, but are not limited to, early identification and diagnosis, prehabilitation, pain management, patient education, non-surgical and post-operative rehabilitation (Boerger et al., 2022b). Current clinical practice guidelines recommend, as an option, structured non-surgical management for individuals with mild DCM and those that are non-myelopathic with spinal cord compression (NMSCC) and concomitant clinical evidence of radiculopathy (Fehlings et al., 2017). Yet, there is a lack of evidence-based care pathways and specific guidance on the content and structure of non-surgical management and rehabilitation for DCM (Boerger et al., 2022b).

Given the frequent diagnostic delays and progressive functional decline seen in DCM, optimizing early diagnosis and non-surgical management to enhance preoperative function and post-operative recovery is critical (Loh et al., 2023). This gap in rehabilitative research and clinical practice has been acknowledged as the sixth key DCM research priority by AO Spine RECODE-DCM (REsearch objectives and COmmon Data Elements for DCM), a global multi-stakeholder research priority setting initiative (aospine.org/recode) (Boerger et al., 2022b). RECODE-DCM involved a multi-stakeholder community working to accelerate the creation and translation of knowledge in DCM. The community is now hosted by Myelopathy.org, a DCM Charity, and includes a number of parallel projects and initiatives. To help address this and other research priorities, a number of expert working groups have been formed, termed incubators. The RECODE-DCM Peri-Operative Rehabilitation Incubator is an expert working group, made up of professionals from a range of different disciplines from around the world and patients with lived experience.

This narrative review explores the essential roles that non-surgical clinicians play in the multidisciplinary assessment and management of DCM, on behalf of the RECODE-DCM Peri-Operative Rehabilitation Incubator. This article represents their appraisal of the current state of the evidence base, which given its limited nature, draws upon their wider experience of the field and related fields, to showcase the need and opportunity. It includes suggestions of where research should now be targeted.

## 2 Materials and methods

### 2.1 Design

This review employs a non-systematic design with narrative synthesis, focusing on key non-surgical DCM management domains identified by the RECODE-DCM Peri-Operative Rehabilitation Incubator, an expert multidisciplinary working group hosted by Myelopathy.org. Given the absence of sufficient literature evaluating the various aspects of non-surgical clinician involvement in DCM (Boerger et al., 2022b), a systematic review was not feasible. Instead, this review synthesizes key concepts identified by the expert incubator, emerging DCM research while also incorporating relevant literature from other related spinal conditions, where necessary.

### 2.2 Definition and scope

**Fig. 1:**
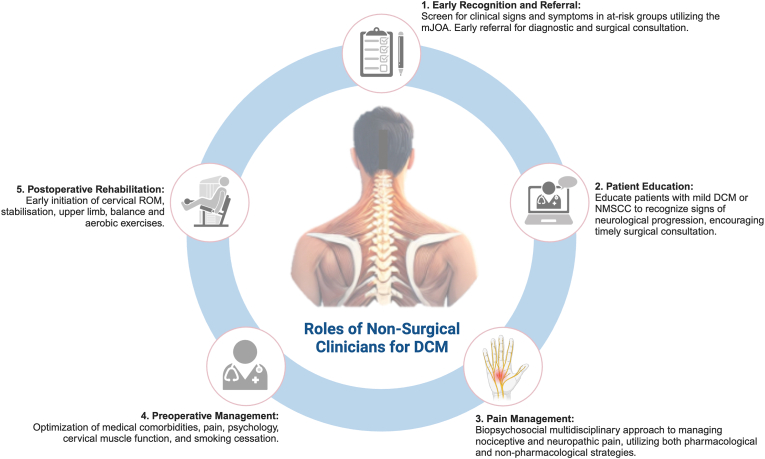
**Key Roles of Non-Surgical Clinicians for Degenerative Cervical Myelopathy**. DCM - Degenerative Cervical Myelopathy; mJOA – modified Japanese Orthopaedic Association score; NMSCC – non-myelopathic spinal cord compression; ROM – range of motion.

This review defines and provides recommendations for the role of non-surgical clinicians in optimizing the health and outcomes of people living with DCM (plwDCM) in the preoperative and postoperative stages. The roles of clinicians in the peri-operative stage, particularly in the hospital level of care has not been included in this review. Non-surgical clinicians play a crucial role across various stages of the patient journey, including early recognition and patient education in the primary healthcare sector, as well as pain management, preoperative health optimization, preoperative and postoperative rehabilitation in the secondary and tertiary care settings (Fig. 1). These were the concepts broadly agreed upon by the RECODE-DCM Peri-Operative Rehabilitation Incubator.

These non-operative clinicians include, but are not limited to, primary care physicians, pain and musculoskeletal medicine physicians, rheumatologists, physical medicine and rehabilitation physicians, neurologists, nurse practitioners and allied health clinicians, such as physical therapists, occupational therapists, osteopaths, and chiropractors. In addition to synthesizing existing literature, expert insights from the RECODE-DCM Peri-Operative Rehabilitation Incubator informed the key concepts for this paper, providing a multidisciplinary perspective in contextualising the available evidence, while identifying gaps in knowledge and clinical practice.

### 2.3 Literature review

To identify relevant peer-reviewed literature on the role of non-surgical clinicians for DCM, we conducted a MEDLINE search using the comprehensive search strategy developed by Davies et al. (2018a). The following additional search terms were applied: “non-surgical” OR “non-operative” AND “rehabilitation” OR “management,” “profession,∗” OR “clinician” to capture articles published between January 1, 2000, and September 1, 2024.

The retrieved articles were reviewed for relevance to the topic of non-surgical management and clinician roles in DCM, with selection criteria favouring studies that discussed early diagnosis, rehabilitation, pain management and patient education aspects within this context. Only articles published in English and appearing in peer-reviewed journals were considered. Where dedicated DCM research was lacking, evidence from related spine conditions (e.g., spinal cord injury, chronic pain syndromes, degenerative spine diseases and elective cervical spine surgery) were reviewed and considered.

### 2.4 Evidence appraisal

The articles included in this narrative review were appraised for their relevance and contribution to understanding the role of non-surgical clinicians in the assessment and management of DCM. The appraisal process focused on identifying key concepts related to non-surgical involvement, including early recognition and referral, patient education, pain management, preoperative management and postoperative rehabilitation.

Given the non-systematic nature of this review, the appraisal emphasized recent articles with clinical applicability rather than formal quality assessment tools. As a result, this review does not include a structured risk-of-bias assessment, which is acknowledged as a limitation. However, the inclusion of expert consensus aims to provide a pragmatic overview of non-surgical management strategies while identifying areas where high-quality, systematic evidence is lacking. The appraisal findings informed the organization of the review topics and highlighted areas where additional research and clinical guidelines are needed.

## 3 Early recognition and referral

Although this paper focuses on non-surgical management, the early recognition of DCM by non-surgical clinicians is crucial to addressing the diagnostic delays commonly faced by plwDCM, which average 2.5 years (Behrbalk et al., 2013). Studies highlight low awareness and diagnostic confidence among primary care providers internationally, particularly general medical practitioners, contributing significantly to these delays (Chauhan et al., 2025; Rufus-Toye et al., 2024; Waqar et al., 2020). Consequently, many individuals with DCM remain undiagnosed (Grodzinski et al., 2023), and are only identified at which point they develop symptoms severe enough to warrant emergency or acute presentations. Reducing diagnostic delays is a priority outlined by RECODE-DCM, as longer symptom durations and greater severity at presentation are associated with poorer outcomes (Davies et al., 2022; Pope et al., 2020).

In the earlier stages of DCM, patients often present with subtle and nonspecific symptoms, requiring greater clinician awareness to consider DCM in patients with subtle deficits such as hand clumsiness or poor balance, rather than dismiss them as part of an aging process (Jiang et al., 2023). While orthopaedic spine surgeons and neurosurgeons manage the majority of plwDCM, patients with early and undiagnosed DCM may present to a variety of clinicians including pain medicine physicians, rheumatologists, neurologists, general orthopaedic and neurological surgeons alongside primary care and allied health clinicians prior to the diagnosis being confirmed. Raising awareness across disciplines is critical, encouraging clinicians to proactively screen at-risk populations for hallmark signs of DCM (Davies et al., 2022).

At-risk groups include individuals with lumbar degenerative conditions such as lumbar spinal stenosis (Overley et al., 2017), older adults (Smith et al., 2021), and those with a history of cervical trauma, such as retired contact-sport athletes (Anderson et al., 2024). Additionally, those having undergone prior cervical spine surgery or those with congenital conditions such as Klippel-Feil syndrome (Nouri et al., 2015b) and Ehlers-Danlos syndrome (Baucher et al., 2022) are also established at-risk groups.

In the absence of validated diagnostic criteria, tools such as the modified Japanese Orthopaedic Association score can help clinicians identify key symptoms (Treanor et al., 2024). Awareness of referral pathways for cervical spine MRI and surgical consultation are equally important to ensure timely diagnosis and intervention (Davies et al., 2022). Long wait times for diagnostics and consultations often exacerbate symptom progression (Pope et al., 2020; Fehlings et al., 2013). In some healthcare systems, advanced practice clinicians assist with the clinical triage of orthopedic and neurosurgical cases, addressing the disparity between demand and service capacity (Williams et al., 2019). Such initiatives may improve the timeliness of diagnosis and treatment for plwDCM.

## 4 Patient education

Patient education is essential for improving awareness of DCM among both patients and family members, enhancing engagement with treatment options, and enabling patients, especially those with mild DCM or asymptomatic cord compression, to recognize early signs of disease progression (Jiang et al., 2023). Timely surgical reassessment is crucial to facilitate early intervention and prevent the adverse outcomes associated with delayed surgical decompression (Davies et al., 2018b). Studies reveal that up to 62 % of untreated DCM patients experience neurological decline over a three-to six-year period, with 75 % exhibiting sudden, unpredictable stepwise neurofunctional deterioration (Wilson et al., 2013; Karadimas et al., 2013). Routine monitoring also shows that 8 % of patients with NMSCC develop symptomatic DCM within one year, increasing to 23 % at four years (Fehlings et al., 2013).

Clinical practice guidelines recommend serial monitoring for patients with mild DCM or NMSCC to ensure early detection and timely intervention (Fehlings et al., 2017). While there is limited evidence to define optimal follow-up intervals, suggested protocols include follow-up every 3–6 months with repeat neuroimaging during the first two years, followed by 6–12 monthly follow-ups for patients who are neurologically and radiologically stable (Furlan, 2023). Expedited surgical consultation is imperative when neurological or functional decline is detected (Fehlings et al., 2017). Ongoing research through the RECODE-DCM Natural History Incubator seeks to refine follow-up protocols. The use of validated tools such as the modified Japanese Orthopaedic Association (mJOA) score (Fehlings et al., 2017), hand-held dynamometry, and gait assessments supports early detection of functional decline and informs surgical decision-making (Loh et al., 2023).

Although surgeons are primarily responsible for counselling patients on surgical approaches, recovery, and postoperative precautions (Fehlings et al., 2017), non-surgical clinicians play an essential complementary role in patient education. They can guide patients and their families in recognizing and monitoring disease progression, adopting lifestyle and occupational modifications, symptom management, and engaging with rehabilitation programs (Furlan, 2023). In particular, patients should be educated on precautions such as avoiding cervical manipulations and implementing fall-prevention strategies to reduce the risk of acute spinal cord injuries, such as central cord syndrome (Wilson et al., 2013).

Non-surgical clinicians also provide critical psychosocial support, helping patients navigate the emotional and practical challenges of living with DCM. Additionally, they can connect patients to support networks, such as the Myelopathy.org Facebook group, which offers both educational resources and psychological support to plwDCM and their families (Davies et al., 2018b).

## 5 Pain management

Pain is a significant symptom for plwDCM, profoundly impacting their quality of life (Boerger et al., 2022a). Pain in DCM arises from multiple mechanisms, including nociceptive somatic inputs from cervical spondylosis and musculature, neuropathic processes affecting the somatosensory system, and nociplastic changes linked to alterations in central nervous system structures (Treede et al., 2008, 2019; Fard et al., 2024).

Pain is the number one recovery priority for plwDCM irrespective of functional status (Davies et al., 2019), with over 50 % of plwDCM experiencing high-impact chronic pain (Cook et al., 2022), particularly neck and arm pain (Davies et al., 2019; Jiang et al., 2023). While surgery addresses spinal cord compression, it may not fully alleviate axial neck pain, highlighting the essential role of non-surgical clinicians (Wang et al., 2011). These clinicians contribute to pain management through pharmacological and non-pharmacological strategies, addressing nociceptive and neuropathic components while incorporating psychosocial support to mitigate chronic pain's broader impacts.

Contemporary evidence in pain management emphasizes a biopsychosocial approach to address the multifaceted pain experienced by plwDCM (Boerger et al., 2022a; Loh et al., 2023; Lee and Kim, 2023). Psychological distress is a key predictor of residual postoperative neck pain and disability in patients undergoing anterior cervical discectomy and fusion (Goh et al., 2019). Other predictors of persistent high-impact pain include preoperative depression, female sex, low baseline mJOA scores, and severe preoperative neck and arm pain (Cook et al., 2022). These findings highlight the importance of holistic pain management strategies across non-surgical, preoperative, and postoperative contexts.

Comprehensive pain assessment should incorporate psychosocial factors alongside traditional measures such as visual analogue scales and numeric pain rating scales (Loh et al., 2023). Tools like the Pain Self-Efficacy Questionnaire (Kondo et al., 2023) and the Hospital Anxiety and Depression Scale provide valuable insights into psychological contributors to pain and disability (Menon et al., 2016). These assessments guide non-surgical clinicians in tailoring interventions for DCM-related pain.

A multimodal pain management strategy should integrate both pharmacological and non-pharmacological approaches (Rhee et al., 2013). Pharmacological options may include non-steroidal anti-inflammatory drugs, opioids, gabapentinoids, tricyclic antidepressants, serotonin-noradrenaline reuptake inhibitors and epidural or zygapophyseal injections (Cohen and Hooten, 2017; Moulin et al., 2014), though evidence supporting their effectiveness in DCM-specific pain is limited (Rhee et al., 2013; Manzur et al., 2023). While cervical epidural injections may provide short-term pain relief in cases of myeloradiculopathy, caution is advised with cervical epidural and zygapophyseal injections, as they are linked to an increased risk of subsequent DCM-related surgery within one year (Manzur et al., 2023).

Non-pharmacological interventions, while not DCM-specific, can be adapted from persistent neck pain management strategies, including cervical traction, massage, therapeutic exercise, heat therapy, acupuncture, transcutaneous electrical nerve stimulation and mindfulness techniques (Childress and Becker, 2016; Cohen and Hooten, 2017). Persistent postoperative neuropathic pain, well-documented in individuals suffering from lower back pain (Alshammari et al., 2023), may benefit from biopsychosocial management approaches such as the Explain Pain framework (Moseley and Butler, 2015), Acceptance and Commitment Therapy (Feliu-Soler et al., 2018), and mindfulness-based interventions alongside physical interventions.

Allied health clinicians, including physical therapists, psychologists, occupational therapists, and pain medicine specialists, are well-positioned to delivering these interventions within a multidisciplinary framework. Their role extends to supporting patients in living well despite persistent pain and optimizing quality of life.

## 6 Preoperative management

The role of preoperative management, including medical optimization and prehabilitation, in improving post-operative outcomes for plwDCM remains largely underexplored in current literature and guidelines (Boerger et al., 2022b; Fehlings et al., 2017). Evidence from elective spine surgery highlights the importance of a multidisciplinary approach to optimizing modifiable risk factors and medical comorbidities. These include endocrine, cardiovascular, and renal health, bone quality, frailty, cervical muscle function, nutrition, obesity, pain management, psychological well-being, and smoking cessation (Wang et al., 2021). Specific to DCM, advanced age, greater disease severity, gait impairment, and smoking are significant predictors of post-operative outcomes, with smoking cessation being particularly imperative in reducing post-operative complications such as non-union and infection (Tetreault et al., 2015).

Preoperative patient education, in the form of a structured 60-min class supplemented by written material, has demonstrated a significant reduction in hospital length of stay in patients undergoing lumbar and cervical fusion surgery (Edwards et al., 2022). Interventions as such may help to normalize expectations regarding surgery and recovery, supporting post-operative outcomes. Individualized prehabilitation programs can further enhance readiness for surgery by addressing muscle dysfunction, functional limitations, fall prevention, and general symptom management (Loh et al., 2023).

Muscle dysfunction, particularly fatty infiltration observed in cervical extensor muscles on axial MRI sequences, is common among plwDCM and correlates with greater disease severity, greater canal compromise, and lower functional scores preoperatively (Fortin et al., 2018; Naghdi et al., 2024). Higher cervical lean muscle mass is associated with improved muscle strength in these patients (Fortin et al., 2018). Recent multi-centric research also indicates that lower cervical muscle function and greater cervical muscle asymmetry are linked to poorer clinical outcomes, including function, disability, and quality of life at 6 and 12 months post-surgery (Naghdi et al., 2023). Targeted prehabilitation exercises focusing on muscle strengthening and neuromuscular conditioning are therefore key clinical variables, and may enhance surgical outcomes and overall recovery.

Given the functional impairments seen in plwDCM, clinicians should incorporate aerobic and resistance exercises, balance training, and gait-specific interventions to mitigate risks, particularly falls, which can lead to severe complications like fractures or acute spinal cord injury (Boerger et al., 2022c; Horowitz et al., 2019). The use of walking aids should be considered on an individual basis to reduce falls risk. Although direct evidence for targeted balance training in DCM is lacking, clinical reasoning supports its inclusion, given the potential for catastrophic outcomes. Interventions may include trunk and limb postural exercises and reactive balance strategies to enhance stability (Furlan, 2023).

## 7 Post-operative rehabilitation

Although surgical decompression is the recommended intervention for moderate to severe or neurologically progressive DCM (Fehlings et al., 2017), up to 95 % of plwDCM experience chronic disability post-surgery (Boerger et al., 2022b; Fehlings et al., 2013). Unlike other neurological conditions such as stroke, traumatic brain injury, or spinal cord injury, standardized post-operative rehabilitation for DCM is neither common nor well-researched (Badran et al., 2018; Boerger et al., 2022b). Given the shared clinical features and impairments, rehabilitation strategies used in these conditions may provide a foundation for managing DCM (Loh et al., 2023).

An individualized multidisciplinary approach is essential, tailored to patient needs and rehabilitation goals (Loh et al., 2023). Recent evidence suggests that occupational- or physical-therapy focusing on cervical range of motion and stabilization within six weeks post-surgery yields the greatest improvements in physical function at 12 months, though no differences were observed in neck disability index scores (Rahman et al., 2024). Critically, this retrospective review did not discuss intensity, frequency, or sub-type of lower extremity physical therapy (Rahman et al., 2024). Standardized rehabilitation programs, encompassing goal setting, medical complication management, targeted strength and range of motion exercises, psychological intervention, and periodic functional and neurological monitoring, have been shown to enhance daily functioning in plwDCM beyond the benefits of surgery alone (Catz et al., 2024).

Recent years have seen a push towards high-intensity locomotor training in patients with stroke and traumatic spinal cord injury (Boerger et al., 2022b). While small trials have shown mixed results (Cheng et al., 2020; Magagnin et al., 2010), significant improvements in gait speed and time to complete the Timed Up and Go test were reported in one study (Cheng et al., 2020). Further advances may include combining rehabilitation with brain and/or spinal cord stimulation (Gharooni et al., 2021, 2022). A recent prospective clinical trial found that repetitive transcranial magnetic stimulation paired with rehabilitation showed greater functional gains compared to standard rehabilitation alone in the first six months postoperatively (Farrokhi et al., 2024). Critically, this clinical trial demonstrated that ASIA, mJOA, Ashworth, and Nurick scores all improved in both groups (with and without stimulation) (Farrokhi et al., 2024). While both groups had improved function, the degree of improvement was indeed greater in those who received stimulation (Farrokhi et al., 2024). These findings demonstrate importantly, that 1) the provision of standard of care rehabilitation indeed increases function in plwDCM and 2) non-invasive stimulation may further maximize benefits of therapy beyond that of standard of care.

Targeted interventions addressing balance deficits (Boerger et al., 2022c, 2024) are critical due to the risk of falls and fragility fractures in this population (Horowitz et al., 2019), particularly since lower extremity recovery is less robust than upper extremity recovery (Friesen et al., 2024). High-intensity, prolonged programs to improve manual dexterity have shown promise but remain underutilized and under-researched in DCM rehabilitation (Loh et al., 2023). Comprehensive post-operative care should also address secondary complications, pain, and psychological distress to optimize outcomes (Loh et al., 2023).

## 8 Research gaps

The RECODE-DCM initiative has played a pivotal role in identifying key research priorities to advance clinical practice and improve outcomes for people living with DCM (plwDCM). However, it has also highlighted significant gaps, particularly regarding the provision and efficacy of nonoperative management for NMSCC and pre- and post-operative rehabilitation for DCM (Boerger et al., 2022b). To address these gaps, prospective controlled studies are needed to delineate the benefits of non-surgical management, with a specific focus on standardizing rehabilitative modalities, determining optimal dosage, intensity, and the need for supervised versus unsupervised elements (Boerger et al., 2022b).

Developing effective rehabilitation programs for DCM should integrate the perspectives of plwDCM to ensure a patient-centred approach that aligns with their needs and preferences (Plener et al., 2024). While rehabilitation programs must remain flexible to accommodate individual variability, data on specific modes and dosages of rehabilitation would enable the creation of evidence-based care pathways and standardized practice guidelines, that can inform global practice changes. Importantly, the implementation of such care pathways depends on the availability of funding and appropriately trained clinicians. Consequently, developing sustainable and actionable rehabilitation models is crucial, both to enhance patient outcomes and to provide robust evidence for stakeholders to support the integration of structured, evidence-based non-surgical interventions alongside surgical care.

Additionally, long-term prospective studies are necessary to deepen understanding of the natural history of DCM. Such studies could inform evidence-based guidelines for follow-up and serial monitoring of individuals with NMSCC or mild, non-progressive DCM undergoing nonoperative management. These efforts are essential for refining care protocols and ensuring timely intervention when needed.

## 9 Limitations

This review has several limitations. First, the non-systematic design may introduce selection bias, as studies were chosen based on thematic relevance rather than predefined inclusion/exclusion criteria. Second, while certain domains (e.g., postoperative rehabilitation, early recognition) have emerging evidence, other domains such as preoperative management, patient education, and pain management, lacked DCM-specific research, necessitating extrapolation from other related spinal conditions. This limits the direct applicability of some findings. Third, the absence of a formal critical appraisal process may affect the robustness of individual study interpretations. Finally, while expert insights provided a valuable framework, they inherently reflect current clinical perspectives rather than systematic evidence synthesis. Despite these limitations, this review provides a broad overview of the key role of non-surgical clinicians in DCM, highlighting critical gaps that warrant further research.

## 10 Conclusions

Non-surgical clinicians play a pivotal role in the assessment and management of plwDCM. This narrative review synthesizes current knowledge, highlighting their contributions across key domains, including early recognition, patient education, pain management, preoperative optimization, and postoperative rehabilitation. Across these domains, a multidisciplinary and individualized, patient-centred approach remains integral. While the potential for postoperative rehabilitation in DCM management is increasingly recognized, significant gaps persist in understanding optimal modalities, dosage, and clinical delivery strategies. Addressing these gaps through robust research and standardized care pathways is essential to enhancing the impact of non-surgical DCM management and ultimately improving outcomes and quality of life for plwDCM.

## Author contributions

RVC: conceptualisation, investigation, writing - original draft preparation, reviewing and editing.

AKD: conceptualisation, writing - reviewing and editing.

TFB: investigation, writing - reviewing and editing.

JML: writing - reviewing and editing.

CT: writing - reviewing and editing.

SKR: writing - reviewing and editing.

VK: writing - reviewing and editing.

LW: writing - reviewing and editing.

JP: writing - reviewing and editing.

NW: writing - reviewing and editing.

MF: writing - reviewing and editing.

CA: writing - reviewing and editing.

AP: writing - reviewing and editing.

RSD: writing - reviewing and editing.

BD: writing - reviewing and editing.

MGF: writing - reviewing and editing.

DBA: conceptualisation, writing - reviewing and editing.

## Ethical approval

Ethical approval was not required for this paper.

## Funding

This research did not receive any specific grant from funding agencies in the public, commercial, or not-for-profit sectors. The authors have no other financial disclosures to declare.

## Declaration of competing interest

The authors have no relevant financial not subject-related conflicts of interest to disclose.
